# Oriented Immobilization and Quantitative Analysis Simultaneously Realized in Sandwich Immunoassay via His-Tagged Nanobody

**DOI:** 10.3390/molecules24101890

**Published:** 2019-05-16

**Authors:** Li Xu, Hanyu Cao, Chundong Huang, Lingyun Jia

**Affiliations:** Liaoning Key Laboratory of Molecular Recognition and imaging, School of Bioengineering, Dalian University of Technology, No.2 Linggong Road, Dalian, Liaoning, 116023, China; xu-li@dlut.edu.cn (L.X.); caohanyujie@163.com (H.C.); cd_huang_bio@mail.dlut.edu.cn (C.H.)

**Keywords:** sandwich immunoassay, nanobody, His-tag, oriented immobilization, β amyloid detection

## Abstract

Despite the advantages of the nanobody, the unique structure limits its use in sandwich immunoassay. In this study, a facile protocol of sandwich immunoassay using the nanobody was established. In brief, β amyloid and SH2, an anti-β amyloid nanobody, were used as capture antibody and antigen, respectively. The SH2 fused with His-tag was first purified and absorbed on Co^2+^-NTA functional matrix and then immobilized through H_2_O_2_ oxidation of Co^2+^ to Co^3+^ under the optimized conditions. Then, 150 mM imidazole and 20 mM EDTA were introduced to remove the unbound SH2. The immobilized SH2 showed highly-sensitive detection of β amyloid. It is interesting that the quantification of the sandwich immunoassay was carried out by determining the His-tag of the detection nanobody, without interference from the His-tag of the capture nanobody. The immobilized SH2 detached exhibited outstanding stability during 30 days of storage. Taken together, His6-tag facilitated both the oriented immobilization of capture antibody and quantitative assay of detection antibody in sandwich immunoassay. We propose a facile and efficient sandwich immunoassay method that opens new avenue to the study of His-tagged protein interactions.

## 1. Introduction

Immunoassay is widely used for the quantitative and/or qualitative analysis of antigens based on the interactions between antibodies and antigens [1,2]. Since it was first applied to detect insulin in the serum using 125I as tag by Yallow and Berson in 1959, immunoassay has been playing vital roles in drug screening, toxicology monitoring, biological, clinical, chemical and environmental analyses because of its high sensitivity and selectivity, as well as rapid detection and simple analysis achieved without extensive pretreatment [3]. Although the radioimmunoassay method is highly reliable and accurate, its use has been limited by radioisotopes and the short half-life [4,5]. To overcome the limitations, intensive research in immunoassay techniques has resulted in tremendous advancement. For example, to easily quantify results of immunoassay, antigens or antibodies were modified by different luminescent substances or enzymes [6,7]. To keep consistency between different batches, monoclonal antibodies are most widely used. The traditional monoclonal antibody IgG consists of two heavy chains and two light chains. Because protein fragments are more amenable to engineering and production, the hunt for the smallest antibody fragment capable of binding to antigens continues and progressions such as 55-kDa Fab and 28-kDa scFv have been made. However, problems remain in the expression and aggregation of these antibodies [8,9]. A distinct type of antibody, which contains only the heavy chain, was discovered in the serum of alpaca, camel and cartilage fish in 1993 [10]. Following the discovery came the nanobody, a 15 kDa antibody fragment derived from the heavy-chain-only antibody. Compared with the traditional antibodies and their fragments, nanobodies exhibit superior characteristics in many aspects, such as the ease in screening and preparation, high sensitivity, stability, specificity, and the low steric hindrance during the interactions with antigens [11,12,13,14]. Given these advantages, the nanobody greatly improves the immunoassay process.

In sandwich immunoassay, the capture antibody is usually randomly immobilized or coated on materials based on the hydrophobic interaction. Since every domain of the nanobody is vital in either antigenic binding or structural maintenance, random immobilization will lead to the inactivation of the nanobody. Thus, it is important to find a suitable immobilization method for the capture nanobody. Moreover, the constant region of the nanobody, which can be recognized by the second antibody, needs to be exposed for the quantitative detection. The easiest way to immobilize proteins is to limit the modifications within the region not involved in the interactions of antigen. Immobilized metal ion affinity chromatography (IMAC) mediated by a fused poly-histidine tag (His-tag) has been used as a simple and inexpensive purification technique for the high-throughput production of proteins in industry [15,16,17]. IMAC has also been extended to various other applications, such as the immobilization of proteins on chips and nanomaterials [18,19,20], and the incorporation of labeled molecules to active proteins [21,22,23,24]. Divalent ions such as Ni^2+^, Co^2+^, Cu^2+^ and Zn^2+^ can mediate the chelation between the His-tag and nitrotriacetic acid (NTA) [14,15]. However, the aforementioned affinity is not stable enough in buffer containing chelating or reducing reagent. Seraphine and others reported that Co^2+^ in the complex of NTA-Co^2+^-His-tagged protein could be converted to Co^3+^ through H_2_O_2_ oxidation [25]. The complex of NTA-Co^3+^-His-tagged protein is thermodynamically stable, and it reacts weakly with chelating or reducing agents. In consistence, we recently reported that the BirA enzyme showed excellent stability and catalytic activity using the same immobilization method [26]. Given the short reaction time and the stability after long-term storage, we postulated the method may serve as a facile approach for the oriented immobilization of the capture antibody in the immunoassay. Compared to the random immobilization, oriented immobilization via NTA-Co^3+^-His-tagged complex will not affect the conformation or the function of nanobody. To further improve the application of nanobody in the sandwich immunoassay, we propose the use of His-tag as both the immobilization site of the capture antibody and the recognition site for the second detection antibody. However, in practical research, the His-tag is often designed on both the capture antibody and the detection antibody. To realize our postulation, the interaction between the second antibody and the His-tag of the capture antibody should be avoided in sandwich immunoassay indicating the process of sandwich immunoassay should be accordingly optimized.

Alzheimer’s disease (AD) is a neurodegenerative disease associated with aging. Soluble β-amyloid (Aβ) is believed to be a contributing factor in Alzheimer’s disease. It is well documented that the changes of Aβ level in simulated cerebrospinal fluid (CSF) may indicate the occurrence of early AD [27,28]. Therefore, soluble β-amyloid and anti-Aβ nanobodies were selected as model antigen and antibodies in our study. Conditions of immobilizing the nanobody to the NTA-Co^3+^ were first optimized. The activity and the stability of the immobilized nanobody were then measured. Because Aβ oligomers (Aβo) have more than one of the same antigenic epitopes in one complex, one type of His-tagged nanobody is enough to carry out sandwich immunoassay, while two different His-tagged nanobodies, which recognize different antigenic epitopes, are needed to assay Aβ monomer. Our results show oriented immobilization of nanobody and sandwich immunoassay of Aβ monomer or Aβo could be simultaneously realized only using His-tag nanobody. The advantages of our method are that it is simple and economical compared to the traditional immunoassay method. Certainly, the limitation of this method is that it can only be used in ELISA, which requires sandwich detection. Moreover, we suggest that the method can also be applied to research the interaction molecules recognized by His-tagged proteins.

## 2. Results and Discussion

The most important postulation of our method is that the second antibody, which recognizes the His-tag of the detection antibody, does not bind to the His-tag of the capture antibody. Moreover, oxidation of H_2_O_2_ does not affect the activity of the capture antibodies, while the excess Co^2+^ and unbound capture antibody are removed to improve the interactions of antigen and the detection antibody. The stability of the immobilized capture antibody during storage is also important in application. Thus, we optimized the conditions for oriented immobilization of nanobody and the sandwich immunoassay using His-tags. Nanobodies SH2 and SDP6 were fused with His-tag and selected as model antibody in our study. SH2, which recognizes the N-terminal of Aβ, was immobilized and used as the capture antibody. To detect Aβo, SH2 was also used as detection antibody, while Aβ12-35 recognizing SDP6 was used as the detection antibody to detect Aβ monomer.

### 2.1. Expression and Purification of the Nanobodies

**Figure 1:**
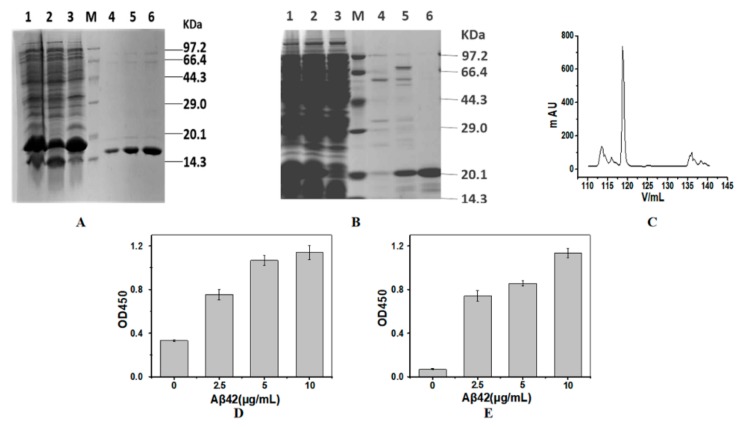
Preparation of the nanobodies. SDS-PAGE analysis of purified SDP6 (**A**) and SH2 (**B**,**C**) by Ni-NTA affinity chromatography (**A**,**B**) and gel chromatography (**C**). Activities of SDP6 (**D**) and SH2 (**E**) were detected by ELISA. Lane M, protein molecular weight marker; Lane 1, Total proteins expressed by *E.coli*; Lane 2, precipitated proteins after the cells were disrupted and centrifuged; Lane 3, supernatant proteins after the cells were disrupted and centrifuged; Lanes 4–6 of SDP6 were SDP6 nanobody eluted by 60 mM imidazole; Lanes 4 and 5 were SH2 nanobody eluted by 100 mM imidazole; Lane 6 of SH2 was SH2 nanobody after gel chromatography.

The theoretical molecular weights of SH2 and SDP6 are 18.7 kDa and 15.6 kDa, respectively. Both nanobodies were fused with His-tag and purified through IMAC. SDS-PAGE was used to evaluate the expression and purification of proteins. As shown in Figure 1A,B, SH2 and SDP6 were successfully expressed by *E.coli*. After purification by IMAC, the purity of SDP6 reached 90% (Figure 1A), while the purity of SH2 remained low (Figure 1B). After further gel filtration, the purity of SH2 reached higher than 95% (Figure 1B,C). ELISA results show that the purified SH2 and SDP6 had the ability to bind antigen Aβ42 (Figure 1D,E). Taken together, SH2 and SDP6 were successively obtained and fit for the following research.

### 2.2. Immobilization of SH2

#### 2.2.1. Effects of H_2_O_2_ Oxidation Time and SH2 Concentration on SH2 Immobilization

**Figure 2:**
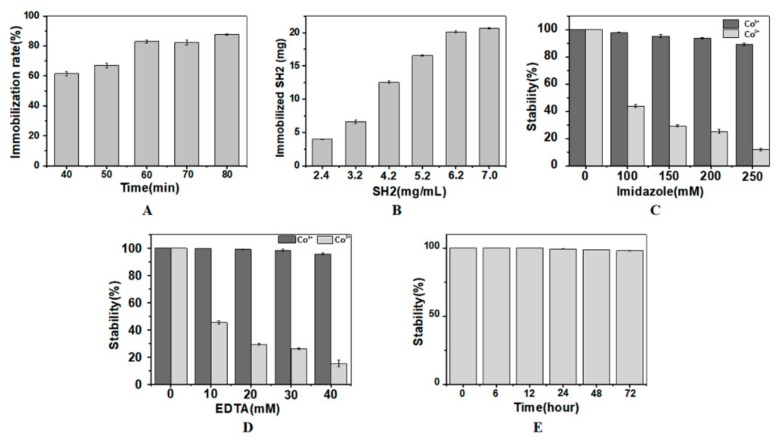
Immobilization of SH2. Optimization of H_2_O_2_ oxidation time (**A**) and SH2 concentration (**B**) for SH2 immobilization. In (**A**), 50 mg SH2 at the loading concentration of 6.2 mg/mL was considered as 100%. Optimization of concentrations and incubation time of imidazole (**C**) and EDTA (**D**,**E**) for the stable immobilization of SH2. In (**A**,**B**), nanobody added in system was considered as 100%. In (**C**,**D**), the amount of immobilized SH2 washed by buffer without imidazole or EDTA was considered as 100%. In (**E**), the amount of immobilized SH2 treated by 150 mM imidazole and 20 mM EDTA for 2 h was considered as 100%. (*n* = 3).

Although His-tagged proteins can bind to NTA as mediated by Co^2+^, the complex of protein-Co^2+^-NTA is not stable enough. After treatment with H_2_O_2_, Co^2+^ is oxidized to Co^3+^, which forms complex of protein-Co^3+^-NTA that is kinetically inert [25,29,30]. However, it is reported that H_2_O_2_ can chemically modify many residues (Met, Cys, Arg, Pro, Lys, Tyr, His, etc.) and cleave the peptide bonds of proteins sustaining the structures and functions of proteins [31]. Met and Cys are two residues particularly sensitive to oxidation [32]. Taking several Cys, Met, Tyr and other residues in the SH2 into consideration, H_2_O_2_ may have negative effects on the nanobody’s structure, activity and stability during immobilization. To investigate whether the activity of SH2 was affected by H_2_O_2_, SH2 was incubated in H_2_O_2_ at the final concentration of 20 mM for different time periods. To remove the unbound SH2 and the excess of Co^2+^, agarose beads were washed with 20 mM EDTA in Tris-HCl buffer (pH 7.4). The concentration of SH2 in washing buffer was measured by the Bradford assay. ELISA assay showed SH2 is not sensitive to H_2_O_2_. The activities of SH2 were not significantly changed in 1 h (data not shown). The immobilization of SH2 improved with the increase of oxidation time (Figure 2A). The immobilization rates increased from 61.4% to 82.9% when the treatment time of H_2_O_2_ increased from 40 min to 60 min. After 60 min, no significant changes were observed as incubation time increased. Taken together, the oxidation time was optimized as 60 min to realize highly efficient immobilization without compromising the catalytic activity of the SH2.

The binding of SH2 and Co^2+^ is a dynamic equilibrium process, increasing the concentration of SH2 promotes the formation of SH2-Co^2+^-NTA and therefore the conversion to SH2-Co^3+^-NTA in the presence of H_2_O_2_. The concentration of SH2 was accordingly optimized to increase the immobilization density and the utilization rate. To remove the unbound SH2 and the excess Co^2+^, agarose beads were washed with 10 mM EDTA and 150 mM imidazole in Tris-HCl buffer (pH 7.4). As shown in Figure 2B, 6.66 mg, 12.53 mg and 20.14 mg of the SH2 were immobilized in 1 mL agarose beads when 3.2 mg/mL, 4.2 mg/mL and 6.2 mg/mL of SH2 were initially added, respectively. However, further increase of SH2 to 7 mg/mL did not cause significant change of immobilization, which is likely due to the saturated adsorption of the matrix. According to the manual of GE healthcare life sciences, saturated adsorption capacity of Ni^2+^-NTA agarose beads is 20–40 mg. Taken together, initial loading concentration of SH2 was optimized as 6.2 mg/mL.

#### 2.2.2. Effects of Imidazole and EDTA Elution on Immobilized SH2

In the process of immobilization, not all SH2 are firmly bonded to Co^2+^, and not all SH2-Co^2+^-NTA get transformed to SH2-Co^3+^-NTA. Free SH2 and/or residual SH2-Co^2+^-NTA may bind with antigen and affect the accuracy of the detection in the following sandwich immunoassay. Imidazole is a competitive chelating agent of the His-tagged proteins. High concentrations of imidazole replace SH2 bound to Co^2+^ and Co3^+^. The interactions between Co^3+^ and the His-tagged protein are stronger than that between Co^2+^ and the His-tagged protein. To remove the loosely immobilized SH2, the concentrations of imidazole in the eluate should be such that the complexes of SH2-Co^2+^-NTA dissociate while the complex of SH2-Co^3+^-NTA remain intact. As shown in Figure 2C, SH2 in the two types of complexes was stable when there was no imidazole. When treated by 100 mM, 150 mM and 250 mM of imidazole for 2 h, the releasing rates of SH2 from the Co^3+^ complex were 2.03%, 4.73% and 11.87%, respectively. On the contrary, in the Co^2+^ complexes, the release rate was 56.17%, 70.73% and 89.10%, respectively. Based on these results, 150 mM imidazole was added into the eluent buffer to remove the unbound SH2.

In our study, the amount of Co^2+^ in the matrix surpasses the SH2 added. Extra Co^2+^ may affect the following interactions between the Aβ and the capture nanobody or the detection nanobody. To avoid the non-specific adsorptions in immunoassay, the excess of Co^2+^ should be removed by chelating reagent in advance. EDTA is the most commonly used chelation reagent in metal chelation chromatography. EDTA reacts with both Co^2+^ and Co^3+^, preferentially with the former. Given excess EDTA in the buffer may strip off the immobilized SH2, its concentration needs to be optimized. To explore the tolerance of SH2-Co^2+^-NTA and SH2-Co^3+^-NTA to EDTA, the concentration of EDTA in the eluent was determined. As shown in Figure 2D, the free SH2 was not detected when the immobilized SH2-Co^3+^-NTA was incubated in buffer containing 10 mM and 20 mM EDTA for 2 h. When EDTA was further increased to 30 mM, only 4.4% of SH2 dissociated. While 54.6%, 73.9% and 84.8% of SH2 was removed from the SH2-Co^2+^-NTA by 10 mM, 20 mM and 30 mM EDTA, respectively. Based on these results, 20 mM EDTA was added into the eluent buffer to remove excess Co^2+^ and unstably immobilized nanobody.

In conclusion, the presence of imidazole and EDTA affected the stability of the immobilized SH2. In addition to the concentration, incubation time of EDTA and imidazole also affected the stability of the immobilized SH2. As shown in Figure 2E, the retention rates of SH2 in the Co^3+^ complex were not significantly changed in 72 h, indicating the immobilized SH2 was stable in buffer containing 150 mM imidazole and 20 mM EDTA.

### 2.3. Sandwich Immunoassay Using the Immobilized Nanobody

Aβo and Aβ42 monomer was used as model antigens in our study. According to the published data, Aβo contains more than one identical epitopes. To detect Aβo, SH2, which could recognize the *N*-terminal of Aβ, was used as both the capture and the detection nanobody. To detect Aβ42 monomer, another His-tagged nanobody SDP6, which can recognize Aβ12–35, was used as the detection nanobody.

**Figure 3:**
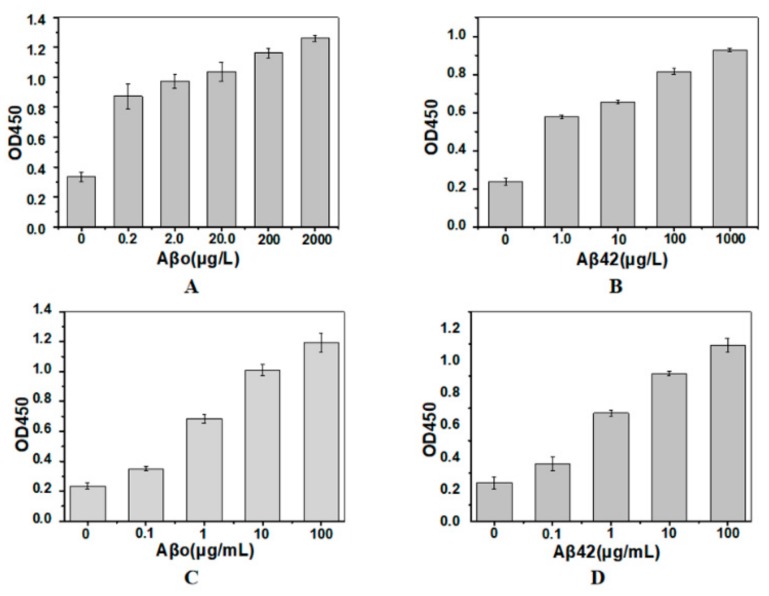
Sandwich immunoassay of Aβo (**A**,**C**) and Aβ42 monomer (**B**,**D**) in simulated cerebrospinal fluid (**A**,**B**) and serum (**C**,**D**). The capture nanobody was the immobilized SH2. The detection nanobodies for Aβo and Aβ42 were His-tagged SH2 and SDP6, respectively (*n* = 3).

Different concentrations of Aβo or Aβ42 monomer dissolved in simulated cerebrospinal fluid (CSF) were incubated with the immobilized SH2. Then, the His-tagged detection nanobody and anti-His-tag monoclonal antibody were incubated sequentially. Sandwich immunoassay results show that the values of OD_450_ were very low when antigen was not added, and increased when antigen was added (Figure 3A), indicating the His-tag of the capture nanobody could not be recognized by anti-His-tag antibody. This is the primary premise to use our method in application. Moreover, the immobilized SH2 could detect a minimum of 0.2 μg/L of Aβo in the SCF, and the value of OD_450_ increased as the concentration of Aβo increased. Similar results were found in the detection of Aβ monomer (Figure 3B). Consistent with results obtained in the SCF, OD_450_ values representing Aβo or Aβ monomer detection in the serum increased with raising antigen concentrations (Figure 3C,D), whereas the sensitivity of the immunoassay dramatically decreased, possibly attributable to the complex composition of serum. Large number of proteins, such as human serum albumin (HSA) [33], apolipoprotein E and apolipoprotein J [34], can bind to Aβ. As the aforementioned proteins are more flexible than the immobilized SH2, their competitive binding with target antigens Aβo and Aβ42, lowered the sensitivity of sandwich immunoassay.

### 2.4. Storage Stability of the Immobilized SH2

**Figure 4:**
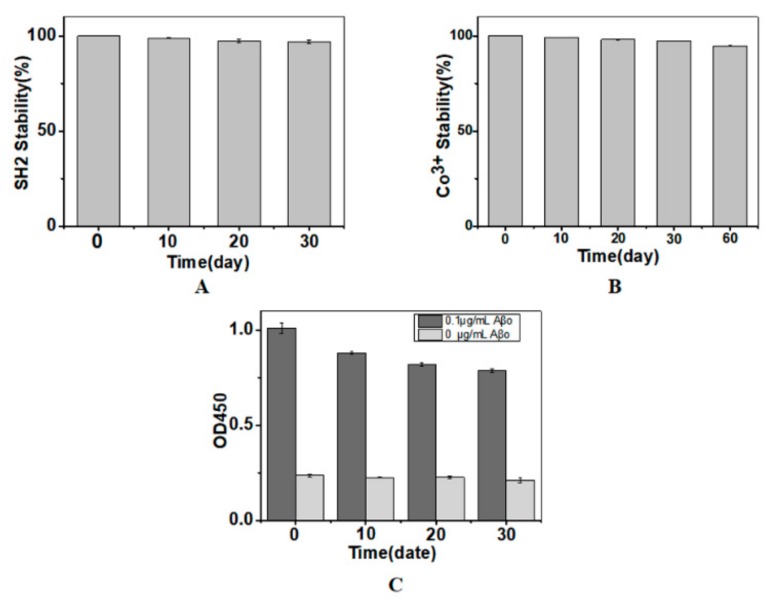
Release of SH2 (**A**); release of Co^3+^ (**B**); and the immuno-activity of SH2 (**C**) during storage. Total amount of the nanobody or Co^3+^ and the immune-activity of the nanobody detected immediately after SH2 immobilization were considered as 100% (*n* = 3).

During storage, the immobilized SH2 might detach from the matrix or lose its bioactivity. To evaluate the stability during storage, the immobilized nanobody was stored in 15 mM Tris-HCl buffer for different time periods. The free nanobody and Co^3+^ in the storing buffer were measured by Bradford assay and inductively coupled plasma optical emission spectrometer, respectively. Total amount of the nanobody or Co^3+^ and the immune-activity of the nanobody detected immediately after SH2 immobilization were considered as 100%. No significant release of nanobody or Co^3+^ was detected when the immobilized SH2 was kept at 4 °C for 10 days. By the end of the assay (30 and 60 days, respectively), no more than 3% of SH2 or Co^3+^ was detected in the storing buffer (Figure 4A,B), indicating the complexes of NTA-Co^3+^-SH2 were stable. However, the activity of SH2 was reduced by 15%, 19.9% and 23.8% (Figure 4C) after being stored for 10, 20 and 30 days, respectively, possibly because of the precipitation of SH2 at 4 °C. Nanobodies with better thermostability can lead to superior performance.

## 3. Materials and Methods

### 3.1. Reagents and Materials

Citric acid, trisodium citrate dehydrate, sodium bicarbonate, sodium carbonate anhydrous and 30% hydrogen peroxide were supplied by Kermal, Tianjin, China. Ethylene diamine tetra-acetic acid disodium, sodium chloride, magnesium acetate and cobalt (II) chloride were provided by J&K Chemical, Beijing, China. Tris, isopropyl-b-thiogalactopyranoside (IPTG), peptone, yeast powder, and BCA protein assays kit and Bradford protein assays kit were supplied by Solarbio, Beijing, China. His Trap FF was supplied by GE Healthcare Life Sciences, Chiltern, Buckinghamshire, UK. All other chemicals were of analytical grade.

### 3.2. Preparation of Aβ Monomer and Aβo

Aβ42 was dissolved in cold hexafluoroisopropanol (HFIP) to a concentration of 1 mM and then sonicated for 10 min. The HFIP was removed by evaporation under a gentle stream of N_2_. Then, the obtained peptide film was stored at −20 °C until use. Aβ42 monomer was obtained by dissolving the peptide film in a small amount of DMSO. For immunoassays, the Aβ monomer solution was diluted with simulated cerebrospinal fluid (containing 125 mM NaCl, 2.5 mM KCl, 0.6 mM NaH_2_PO_4_, 12.8 mM NaHCO_3_, 1 mM MgCl_2_, 2 mM CaCl_2_, and 5 mM glucose, pH 7.4) to a concentration of 25 μM and centrifuged at 4 °C and 11,000 rpm for 10 min. Aβo was prepared by storing the Aβ42 monomer solution at 4 °C for 24 h, followed by centrifugation at 11,000 rpm for 10 min [35]. During assay, Aβ was dissolved in the simulated cerebrospinal fluid or the serum of healthy volunteers accordingly.

### 3.3. Expression and Purification of Nanobodies

Aβ42 was used as an antigen to immunize *Lama pacos* three times. The peripheral blood of *Lama pacos* was used as a material, and T7 phage was used as a display vector. A series of steps such as extract lymphocyte RNA, reverse transcription and in vitro packaging were used to establish an immunogenic library of nanobodies. High affinity and high specificity nanobodies were screened from the original library using phage display technology. Two types of nanobodies named SDP6 (Mr = 15.6 kDa, KD = 4.6 × 10^−7^) and SH2 (Mr = 18.7 kDa, KD = 1.9 × 10^−9^) were prepared in our study. There is a His tag in SDP6. There are a His tag and a Myc tag in SH2. First, genes of His-tagged nanobodies were cloned into the pET-23a using NdeI and XhoI. The recombinant plasmids were then transformed into *E.coli* Shuffle T7. Expressions of the recombinant proteins were induced by 0.25 mM IPTG at 18 °C. After centrifugation, the precipitated cells were suspended in 15 mM Tris-HCl (pH 7.4) containing 500 mM NaCl and 20 mM imidazole and disrupted by sonication. The supernatant was filtered through a HisTrap chelate HP column (GE Healthcare, Chiltern, Buckinghamshire, UK) and eluted with 15 mM Tris-HCl (pH 7.4) containing 500 mM NaCl and 60 mM imidazole. The recombinant proteins were then concentrated in an Amicon ultracentrifugal filter unit (Millipore, USA). Expression and purification of the nanobodies were examined by 15% SDS-PAGE. Activity of the purified nanobody was measured by ELISA. and SDP6

### 3.4. Indirect ELISA to Measure Activity of the Purified Nanobodies

The antigen-binding activities of the purified nanobodies were assayed by ELISA. Aβ was incubated in 96-well plate for 1 h at 37 °C. After incubation, the plate was washed five times with PBS containing 0.05% Tween 20. BSA was used as the negative control. After coating with Aβ42 monomer at different concentrations, the plate was blocked with 2% BSA, followed by the addition of nanobody, anti-His-tag antibody and HRP-conjugated IgG antibody (Sangon Biotech, Shanghai, China). At last, tetramethylbenzidine at 100 mg/L was added to each well and incubated for 20 min before quenching with 2 M H_2_SO_4_. The absorbance was measured at 450 nm using a SUNRISE XFLUOR4 reader (TECAN, Männedorf, Swiss).

### 3.5. Immobilization Nanobody on Nitrilotriacetic Acid Modified Beads

After eluting Ni^2+^-NTA agarose beads (GE Healthcare, USA) with 100 mM EDTA, a Co^2+^ complex with NTA agarose beads was first obtained through 100 mM CoCl_2_ incubation. To determine the optimal concentration of nanobody for the immobilization on agarose beads by 10 mM H_2_O_2_, 2.4 mg/mL, 3.2 mg/mL, 4.2 mg/mL, 5.2 mg/mL, 6.2 mg/mL and 7.0 mg/mL were tested (50 mg nanobody were added into 1 mL agarose beads, followed by 1 h incubation at room temperature) [25]. The ratio of immobilized nanobody to total nanobody was considered as efficiency of immobilization. To remove the unbound nanobody and H_2_O_2_, the agarose beads were eluted with 10 mM Tris-HCl containing different concentrations of imidazole (100 mM, 150 mM, 200 mM, and250 mM) for different time (2 h, 6 h, 12 h, 24 h and 48 h). To remove the excess Co^2+^ and to reduce the non-specific adsorption, agarose beads were eluted with 10 mM Tris-HCl containing different concentrations of EDTA (10 mM, 20 mM, 30 mM and 40 mM). The concentration of the unbound nanobody in the supernatant after each elution was measured by the Bradford assay [36]. Activity of the immobilized nanobody was measured by sandwich ELISA.

### 3.6. Sandwich ELISA

Conditions of the incubation and elution were the same as in the ELISA described above. In brief, different concentrations of Aβ monomer or Aβo were dissolved in the simulated cerebrospinal fluid or serum, and then added into the immobilized nanobody, followed by the addition of the detection nanobody, anti-His-tag antibody, HRP-conjugated IgG antibody and the HRP substrate tetramethylbenzidine. The amount of Aβ was represented by absorbance measured at OD_450_.

### 3.7. Storage Stability of the Immobilized Nanobody

The immobilized nanobody was immersed in 15 mM Tris-HCl buffer and stored at 4 °C for certain periods of time (10, 20 and 30 days). The concentration of free nanobody in the supernatant was measured by Bradford assay. At the same time, the content of free cobalt in the supernatant was determined by flame atomic absorption spectrometry. The activity of the immobilized nanobody after storage was measured by sandwich ELISA.

## 4. Conclusions

**Figure 5:**
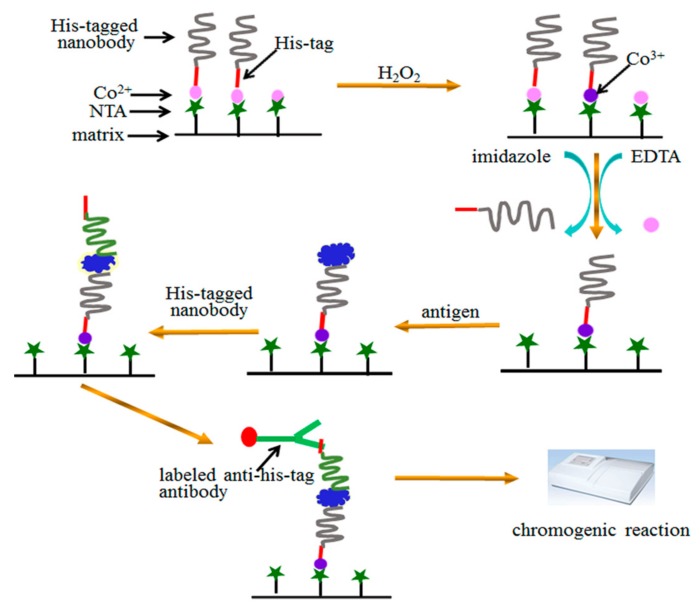
A scheme for oriented immobilization and quantitative analysis in sandwich immunoassay via His-tagged nanobody.

A facile sandwich immunoassay was proposed in our study (Figure 5). Simply, His-tag of the capture nanobody was used to orient immobilization of nanobody on Co^2+^-NTA. Stable complex of Co^3+^-NTA-protein was formed by H_2_O_2_ oxidization. His-tag of the detection nanobody was used to quantify the sensitivity of sandwich immunoassay, which was not affected by the His-tag of the capture nanobody. It is enough to form stable complex of Co^3+^-NTA-protein during 60 min of H_2_O_2_ oxidization. The excess Co^2+^ and unstable SH2 were removed by 20 mM EDTA and 150 mM imidazole, which did not affect the stability of the immobilized Co^3+^-NTA-nanobody. The sensitive detection of A*β* proved the bioactivity of immobilized nanobody while the storage under different conditions confirmed the stability of the proposed assay. Taken together, our method is very good, especially for a nanobody that has only one binding domain and does not have Fc fragment. Moreover, this method holds potential as a useful tool in research investigating the interactions of proteins containing His-tag.

## Abbreviations

NTA	nitrotriacetic acid
scFv	single chain antibody fragment
Aβ	β amyloid
Aβo	oligomer oligomer β amyloid
ELISA	enzyme linked immunosorbent assay

## References

[1] Harlow E., Lane D. (1999). Using Antibodies: A Laboratory Manual.

[2] Shepherd P., Dean C. (2000). Monoclonal Antibodies.

[3] Berson S.A., Yalow R.S. (1959). Quantitative aspects of the reaction between insulin and insulin-binding antibody. J. Clin. Invest..

[4] Surugiu I., Svitel J., Ye L., Haupt K., Danielsson B. (2001). Development of a flow injection capillary chemiluminescent ELISA using an imprinted polymer instead of the antibody. Anal. Chem..

[5] Li Z.P., Wang Y.C., Liu C.H., Li Y.K. (2005). Development of chemiluminescence detection of gold nanoparticles in biological conjugates for immunoassay. Anal. Chim. Acta.

[6] Engvall E., Perlmann P. (1971). Enzyme-linked immunosorbent assay (Elisa) quantitative assay of immunoglobulin-G. Immunochemistry.

[7] Arakawa H., Maeda M., Tsuji A. (1979). Chemi-luminescence enzyme immunoassay of cortisol using peroxidase as label. Anal. Biochem..

[8] Klein C., Hagenah J., Landwehrmeyer B., Münte T., Klockgether T. (2011). The presymptomatic stage of neurodegenerative disorders. Der Nervenarzt.

[9] Huang X., Wang J., Cui L., Zou X., Zhang Y. (2010). Recombinant GST-I-A beta 28-induced efficient serum antibody against A beta 42. J. Neurosci. Methods.

[10] Hamers-Casterman C., Atarhouch T., Muyldermans S., Robinson G., Hamers C., Songa E.B., Bendahman N., Hamers R. (1993). Naturally occurring antibodies devoid of light chains. Nature.

[11] Bruce M.P., Boyd V., Duch C., White J.R. (2002). Dialysis-based bioreactor systems for the production of monoclonal antibodies-alternatives to ascites production in mice. J. Immunol. Methods.

[12] Zheng M.Z., Richard J.J., Binder J. (2006). A review of rapid methods for the analysis of mycotoxins. Mycopathologia.

[13] Luo L., Zhang Z., Hou L., Wang J., Tian W. (2007). The study of a chemiluminescence immunoassay using the peroxyoxalate chemiluminescent reaction and its application. Talanta.

[14] Liu W.S., Song H.P., Chen Q., Yu J.L., Xian M., Nian R., Feng D.X. (2018). Recent advances in the selection and identification of antigen-specific nanobodies. Mol. Immunol..

[15] Hemdan E.S., Zhao Y.J., Sulkowski E., Porath J. (1989). Surface topography of histidine residues: a facile probe by immobilized metal ion affinity chromatography. Proc. Natl. Acad. Sci. USA.

[16] Yip T.T., Hutchens T.W. (1992). Immobilized metal ion affinity chromatography. Protein Expres. Purif..

[17] Kurztkowska K., Mielecki M., Grzelak K., Verwilst P., Dehaen W., Radecki J., Radecka H. (2014). Immobilization of His-tagged kinase JAK2 onto the surface of a plasmon resonance gold disc modified with different copper (II) complexes. Talanta.

[18] Rusmini F., Zhong Z., Feijen J. (2007). Protein immobilization strategies for protein biochips. Biomacromolecules.

[19] Valiokas R., Klenkar G., Tinazli A., Tampe R., Liedberg B., Piehler J. (2010). Differential protein assembly on micropatterned surfaces with tailored molecular and surface multivalency. Chembiochem.

[20] Park S.J., Kim S., Kim S.H., Park K.M., Hwang B.H. (2018). His-tagged protein immobilization on cationic ferrite magnetic nanoparticles. Korean J. Chem. Eng..

[21] Goldsmith C.R., Jaworski J., Sheng M., Lippard S.J. (2006). Selective labeling of extracellular proteins containing polyhistidine sequences by a fluorescein-nitrilotriacetic acid conjugate. J. Am. Chem. Soc..

[22] Kamoto M., Umezawa N., Kato N., Higuchi T. (2010). Novel probes showing specific fluorescence enhancement on binding to a hexahistidine tag. Chem. Eur. J..

[23] Gavutis M., Lata S., Piehler J. (2006). Probing 2-dimensional protein-protein interactions on model membranes. Nat. Protoc..

[24] Pires M.M., Ernenwein D., Chmielewski J. (2011). Selective decoration and release of His-tagged proteins from metal-assembled collagen peptide microflorettes. Biomacromolecules.

[25] Wegner S.V., Spatz J.P. (2013). Cobalt(III) as a stable and inert mediator ion between NTA and His6-tagged proteins. Angew Chem. Int. Edit..

[26] Xu L., Wang R., Cao H.Y., Xu T., Han L.L., Huang C.D., Jia L.Y. (2019). A facile method to oriented immobilization of His-tagged BirA on Co^3+^-NTA agarose beads. Enzyme Microb. Tech..

[27] Racine A.M., Koscik R.L., Nicholas C.R., Clark L.R., Okonkwo O.C., Oh J.M., Hillmer A.T., Murali D., Barnhart T.E., Betthauser T.J. (2016). Cerebrospinal fluid ratios with Abeta42 predict preclinical brain beta-amyloid accumulation. Alzheheimer’s Dement..

[28] Adamczuk K., Schaeverbeke J., Vanderstichele H.M., Lilja J., Nelissen N., Van Laere K., Dupont P., Hilven K., Poesen K., Vandenberghe R. (2015). Diagnostic value of cerebrospinal floid Abeta ratios in preclinical Alzhemer’s disease. Alzhemer’s Res. Ther..

[29] Shao S., Geng J., Ah Yi H., Gogia S., Neelamegham S., Jacobs A., Lovell J.F. (2015). Functionalization of cobalt porphyrin-phospholipid bilayers with His-tagged ligands and antigens. Nat. Chem..

[30] Auer S., Hellmann F., Krause M., Kurths J. (2017). Stable immobilisation of His-tagged proteins on BLI biosensor surface using cobalt. Chaosl.

[31] Hernandez K., Berenguer-Murcia A., Rodrigues R.C., Fernandez-Lafuente R. (2012). Hydrogen peroxide in biocatalysis. Curr. Org. Chem..

[32] Stadtman E.R., Levine R.L. (2003). Free radical-mediated oxidation of free amino acids and amino acid residues in proteins. Amino Acids.

[33] Wang C., Cheng F., Xu L., Jia L.Y. (2016). HSA targets multiple Aβ42 species and inhibits the seeding-mediated aggregation and cytotoxicity of Aβ42 aggregates. RSC Adv..

[34] Strittmatter W.J., Weisgraber K.H., Huang D.Y., Dong L.M., Salvesen G.S., Pericakvance M., Schmechel D., Saunders A.M., Goldgaber D., Roses A.D. (1993). Binding of human apolipoprotein E to synthetic amyloid beta peptide: Isoform-specific effects and implications for late-onset Alzheimer’s disease. Proc. Natl. Acad. Sci. USA.

[35] Jan A., Hartley D.M., Lashuel H.A. (2010). Preparation and characterization of toxic Abeta aggregates for structural and functional studies in Alzheimer’s disease research. Nat. Protoc..

[36] Zor T., Selinger Z. (1996). Linearization of the Bradford protein assay increases its sensitivity: Theoretical and experimental studies. Anal. Biochem..

